# Beclin-1 dependent autophagy improves renal outcomes following Unilateral Ureteral Obstruction (UUO) injury

**DOI:** 10.3389/fimmu.2023.1104652

**Published:** 2023-02-16

**Authors:** Reynold I. Lopez-Soler, Azadeh Nikouee, Matthew Kim, Saman Khan, Lakshmi Sivaraman, Xiangzhong Ding, Qun Sophia Zang

**Affiliations:** ^1^ Section of Renal Transplantation, Edward Hines Jr. VA Hospital, Hines, IL, United States; ^2^ Department of Surgery, Division of Intra-Abdominal Transplantation, Loyola University Chicago Stritch School of Medicine, Maywood, IL, United States; ^3^ Department of Surgery, Burn & Shock Trauma Research Institute; Loyola University Chicago Stritch School of Medicine, Maywood, IL, United States; ^4^ Department of Biology, Loyola University Chicago, Chicago, IL, United States; ^5^ Department of Pathology, Loyola University Chicago Stritch School of Medicine, Maywood, IL, United States

**Keywords:** autophagy, fibrosis, renal transplant, Beclin-1, kidney injury

## Abstract

**Background:**

Interstitial Fibrosis and Tubular Atrophy (IFTA) is the most common cause of long-term graft failure following renal transplant. One of the hallmarks of IFTA is the development of interstitial fibrosis and loss of normal renal architecture. In this study, we evaluated the role of autophagy initiation factor Beclin-1 in protecting against post-renal injury fibrosis.

**Methods:**

Adult male wild type (WT) C57BL/6 mice were subjected to Unilateral Ureteral Obstruction (UUO), and kidney tissue samples were harvested at 72-hour, 1- and 3-week post-injury. The UUO-injured and uninjured kidney samples were examined histologically for fibrosis, autophagy flux, inflammation as well activation of the Integrated Stress Response (ISR). We compared WT mice with mice carrying a forced expression of constitutively active mutant form of Beclin-1, *Becn1^F121A/F121A^*.

**Results:**

In all experiments, UUO injury induces a progressive development of fibrosis and inflammation. These pathological signs were diminished in *Becn1^F121A/F121A^* mice. In WT animals, UUO caused a strong blockage of autophagy flux, indicated by continuously increases in LC3II accompanied by an over 3-fold accumulation of p62 1-week post injury. However, increases in LC3II and unaffected p62 level by UUO were observed in *Becn1^F121A/F121A^* mice, suggesting an alleviation of disrupted autophagy. Beclin-1 F121A mutation causes a significant decrease in phosphorylation of inflammatory STING signal and limited production of IL6 and IFN*γ*, but had little effect on TNF-*α*, in response to UUO. Furthermore, activation of ISR signal cascade was detected in UUO-injured in kidneys, namely the phosphorylation signals of elF2S1 and PERK in addition to the stimulated expression of ISR effector ATF4. However, *Becn1^F121A/F121A^* mice did not reveal signs of elF2S1 and PERK activation under the same condition and had a dramatically reduced ATF level at 3-week post injury.

**Conclusions:**

The results suggest that UUO causes a insufficient, maladaptive renal autophagy, which triggered downstream activation of inflammatory STING pathway, production of cytokines, and pathological activation of ISR, eventually leading to the development of fibrosis. Enhancing autophagy *via* Beclin-1 improved renal outcomes with diminished fibrosis, *via* underlying mechanisms of differential regulation of inflammatory mediators and control of maladaptive ISR.

## Introduction

Currently, all renal transplants have a half-life of 12-14 years. Even though we have made great strides in preventing and treating acute rejection, we have not improved long term graft function in the last 30 years (1). Interstitial Fibrosis and Tubular Atrophy (IFTA) is the most common cause of long-term graft failure and is characterized by the development of interstitial collagen deposition resulting in impaired kidney function and ultimately graft failure (2). There is currently no consensus on the specific causes of IFTA nor is there any effective therapy (2). Understanding the risk factors and steps leading to IFTA will allow us to extend the life of renal grafts and expand our knowledge about chronic kidney disease.

There are many pathways and feedback loops leading to renal fibrosis, an indicator of the ability of tissue to attempt repair following injury to prevent chronic scarring. Transplanted kidneys undergo ischemia/reperfusion (IR) injury followed by recovery during transplantation, infection, and rejection. Repeated bouts of issue hypoxia followed by reperfusion has been shown to be one of the likely pathways leading to renal fibrosis (3–8). These continued injury/repair phases could activate pro-fibrotic pathways and lead graft dysfunction and ultimately graft loss (9).

Autophagy is a conserved cellular process that is activated secondary to cellular stress following ischemia-reperfusion injury resulting in the degradation of cytoplasmic components (10, 11). Various studies have linked autophagy to the development of fibrosis in liver, lung, and heart (12, 13). However, the regulatory mechanisms of autophagy in the development of fibrosis are not fully understood. In kidneys, the role of autophagy has not been clearly elucidated, with some studies hinting at an active role in the development of fibrosis (14).

Transplant literature is bereft on the role of autophagy in post-transplant graft function, with some studies showing protection from graft dysfunction linked to the development of autophagy (15, 16). Autophagy is induced in proximal tubules during kidney injury, as a protective attempt in response to stress condition (17). However, as shown in many published studies, autophagy is a sensitive response that changes dynamically according to various physiological and pathological conditions. While adaptive autophagy provides protective benefits of supporting tubular proliferation and promoting repair in the recovery phase following injury, maladaptive autophagy resulting from dysregulated signals produces deteriorated responses. For example, it was shown that autophagy may promote renal fibrosis through inducing tubular atrophy and decomposition, however, it may also mediate intracellular degradation of excessive collagen and thus prevent fibrosis (18). Given the lack of understanding of the linkage between autophagy and chronic injury, it is important to define the underlying molecular mechanisms of autophagy in renal grafts to develop potential future therapeutic approaches.

Previously, in an animal model study of endotoxemia-induced cardiac dysfunction, we discovered that enhancing autophagy *via* the specific activation of Beclin-1, a universally expressed autophagy initiation factor (19, 20), protects myocardial mitochondria, reduces mitochondria-derived danger-associated molecular patterns (DAMPs), and thus, alleviates inflammation, reduces fibrosis, and improves cardiac performance (21). In fact, specific activation of Beclin-1, either genetically or pharmacologically, significantly improves cardiac performance under the challenge of endotoxemia (21). This study leads us to postulate that the targeted activation of autophagy factors may become an effective therapeutic approach to improve outcomes following renal IR injury. Intriguingly, recent work has shown that enhancing autophagy might minimize damage following IR injury and ultimately improve graft function, in both lens epithelial cells (22) and in kidney tissues (23). In a mouse model of acute kidney injury, a forced expression of a gain-of-function mutant form of Beclin-1, and specifically proximal tubular Beclin-1 expression, resulted in decreased fibrosis 14 days post-injury indicating a possible protective effect against IR injury (24). Additionally, Beclin-1 expression has also been implicated as a potential mediator for Matrix Metalloproteinase (MMP) 2 activity, a key factor in the pathways of renal fibrosis following ischemia-reperfusion injury (25). However, other studies have shown that boosting autophagy may trigger maladaptive signaling pathways indicating the complexity of the role of autophagy in mediating renal injury pathways. In this regard, autophagy was found to coincide with elevated inflammation, apoptosis, and fibrosis in peritoneal membrane from patients with peritoneal dialysis (26), and consistently, down- regulation of Beclin-1 expression was found to associated with prevention of fibrogenesis in a mouse hepatic fibrosis model (27). These data enforce the need for defining the true role of autophagy in the response to renal injury.

Unilateral Ureteral Obstruction (UUO) has been shown to be a surrogate for IR injury as the molecular pathways following UUO leading to fibrosis are similar as those seen during both IR injury and post-transplant renal IFTA (28, 29). In this report, we performed experiments with an aim to characterize the molecular and biochemical relationship between renal IR injury and autophagy using a mouse UUO model. We examine the interactions between autophagy, inflammation and subsequent renal fibrosis following UUO. Further, we evaluate the effects of enhancing autophagy by a forced expression of constitutively active mutant form of Beclin-1. Interestingly, during our investigation, we obtained evidence showing that integrated stress response (ISR), an evolutionarily conserved signaling network that is adaptive to viable physiological conditions (30), may have a strong association with renal autophagy and the development of fibrosis following UUO. Therefore, the pathogenesis of post-injury fibrosis may be modulated *via* alterations in Beclin-1 expression.

## Materials and methods

### Experimental animals

Wild type C57BL/6 mice were obtained from The Jackson Laboratory (Bar Harbor, ME). All animals were conditioned in-house for 5-6 days after arrival with a commercial diet and tap water available at will. A mouse strain carrying a F121A mutation in *beclin-1* (*Becn1^F121A/F121A^*) was previously developed (31, 32).

The animal study was reviewed and approved by Loyola University Chicago institutional animal care and use committee (IACUC) and conformed to the National Research Council’s “Guide for the Care and Use of Laboratory Animals” when establishing animal research standards.

### Mouse model of Unilateral Ureteral Obstruction

UUO was used to induce renal fibrosis. Mice were anesthetized using isoflurane for 5 min and were supplied with isoflurane and oxygen throughout the procedure. Following initial incision, the left ureter was exposed and ligated with a non-absorbable suture. Mice were treated with buprenorphine for analgesia (200 mg/kg body weight) after the procedure. The mice were sacrificed at 72 hours, 1-, 2-, and 3-week post-UUO procedure *via* isoflurane anesthesia followed by cervical dislocation. The kidneys were harvested by snap-frozen in liquid nitrogen and stored at -80^0^C or by fixation in 4% paraformaldehyde (PFA). In experiments, comparisons were made between the left side, injured kidney tissue samples and the right side, unligated (uninjured) controls (33).

### Tissue histology

Tissues collected from mice were fixed in 10% buffered formalin and processed for routine histological examination. Tissue blocks were sectioned to 4 micrometers. Hematoxylin and eosin staining, Masson’s trichrome staining, and chromogenic immunohistochemistry (IHC) staining were performed at Loyola University Histology Core facility on the Ventana Benchmark XT automated immunostaining platform (Ventana Medical Systems, Inc. Tucson, AZ). Prior to staining, the slides were deparaffinized in sequential baths of xylene, transferred to sequential baths of 100% ethanol, followed by sequential baths of 95% ethanol, and then rinsed in deionized (DI) water. Immunohistochemical stains were performed using rabbit polyclonal antibodies against mouse LC3-II (Cell Signaling, Danvers, MA; catalog number 4108), p62 (Abcam, catalog number ab91526), phosphor-STING (Ser366), ATF4, phosphor-PERK (Thr980), and phosphor-EIF2S1 (Ser52) (Thermo Fisher Scientific, Waltham, MA; catalog numbers PA5-105674, PA5-86122, MA5-15033, and 44-728). Detection of the antibody signals was performed using a biotinylated anti-rabbit IgG secondary antibody and streptavidin- horseradish peroxidase (HRP), followed by colorimetric detection using chromogen 3,3′- Diaminobenzidine (DAB). The sections were then counterstained with hematoxylin and mounted under coverslips. Images (each area 1 mm^2^) were captured by an Olympus EX51 microscope (Waltham, MA) and quantitatively analyzed by Image J software as described previously (29, 34). A statistical color model was created based on the histogram of these reserved positive color pixels, and values were then normalized to uninjured control (unligated) mouse kidneys (33). At least ten sections were obtained from three to six animals at each time point, and results were analyzed statistically.

### Preparation of tissue lysates and western blot

When animals were sacrificed, tissues were harvested, washed in PBS, snap clamp frozen, and kept at -80^0^C. Tissue lysates were prepared using tissue protein extraction reagent (Thermo Fisher Scientific, Rockford, IL; catalog number 78510). Protein concentrations were quantified using detergent compatible Bradford assay kit (Thermo Fisher Scientific, Rockford, IL; catalog number 23246). Western blot analysis was performed according to established protocol (21). Briefly, prepared SDS-PAGE protein samples were loaded to and run on 4-20% mini- PROTEAN TGX stain-free precast gels (Bio-Rad Laboratories, Hercules, CA; catalog number 4568091). These stain-free gels contain a proprietary trihalo compound to make proteins fluorescent directly in the gel, allowing the immediate visualization of proteins at any point during electrophoresis and western blotting. Upon finished, protein signals on the gels were transferred to PVDF membranes. Membranes were blocked with 5% nonfat milk-PBS at room temperature for 1 hour and subsequently probed with antibody against LC3A/B and p62 (Cell Signaling, Danvers, MA; catalog number 4108 and 5114). The membranes were then rinsed and incubated with corresponding horseradish peroxidase- conjugated anti-rabbit IgG (Bio-Rad, Hercules, CA; catalog number 170-6515). Antibody dilutions and incubation time were according to manufacturer’s instructions. At the end, membranes were rinsed, and bound antibodies were detected by using SuperSignal West Pico Chemiluminescent Substrate (Thermo Scientific; catalog number 34077).

### Quantitative real-time PCR

Total RNA was extracted from snap-frozen kidney samples using Rneasy Mini kit (Qiagen, Germantown MD; catalog number 74106) according to the vendor’s protocol. The extracted RNA concentration and quality was assessed using a NanoDrop 2000 spectrophotometer (Thermo Fisher Scientific, Waltham, MA), followed by verification of RNA quality using non-denaturing agarose “Bleach” gel (35). Isolated RNA was reverse transcribed using iScript reverse transcription supermix (Bio-Rad Laboratories, Hercules, CA; catalog number 1708841) in the ProFlex PCR system (Thermo Fisher Scientific, Waltham, MA). All real time PCR reactions was performed using Universal SYBR Green supermix (Bio-Rad Laboratories, Hercules, CA; catalog number 1725270) in Quant Studio3 PCR system (Thermo Fisher Scientific, Waltham, MA). Gene target specific primers are listed in **Table 1**. Gene expression levels were normalized using glyceraldehyde-3-phosphate dehydrogenase (GAPDH) as an internal control gene. Relative expression of target genes was determined by the 2^-ΔΔCt^ method. Reactions were performed in triplicate.

**Table 1:** qPCR Primers.

Gene	Forward (5′→3′)	Reverse (5′→3′)
ATF4	ATGATGGCTTGGCCAGTG	CCATTTTCTCCAACATCCAATC
IFN γ	AGAAGTAAGTGGAAGGGCCCAGAAG	AGAAGTAAGTGGAAGGGCCCAGAAG
GAPDH	ACTCCACTCACGGCAAATTC	TCTCCATGGTGGTGAAGACA
TNF-*α*	CCCTCACACTCAGATCATCTTCT	GCTACGACGTGGGCTACAG
IL6	TAGTCCTTCCTACCCCAATTTCC	TTGGTCCTTAGCCACTCCTTC

### Statistical analyses

Sample size was chosen based on our previous studies using animal models (21). Values throughout the report were expressed as means, and values of groups were analyzed using GraphPad (La Jolla, CA) PRISM version 4.0 and compared by ANOVA with Tukey’s multiple-comparison test to determine significant differences between groups. Error bars represent standard error for all calculations. Differences were considered statistically significant when *p* ≤ 0.05, and all samples were tested at least in triplicate.

## Results

### Autophagy pathways are obstructed following UUO and ameliorated in Beclin-1 Gain of Function mutant

In this study, we designed experiments aimed at determining how the status of autophagy flux is regulated following UUO using a previously established mouse model (29). As described, the left kidney was subjected to UUO, and the right-side uninjured kidney served as the proper control. Kidney tissues were recovered 72-hours, 1-, and 3-weeks post-UUO, and the status of autophagy was estimated by immunohistochemistry (IHC) using antibodies against autophagy marker proteins microtubule-associated protein 1A/1B-light chain 3 II (LC3II) and p62/SQSTM1. LC3II is generated during autophagosome formation, one of the initial steps in autophagy. p62 is a polyubiquitin- binding protein that is degraded by lysosomes, which is a later stage of autophagy flux (36).

**Figure 1:**
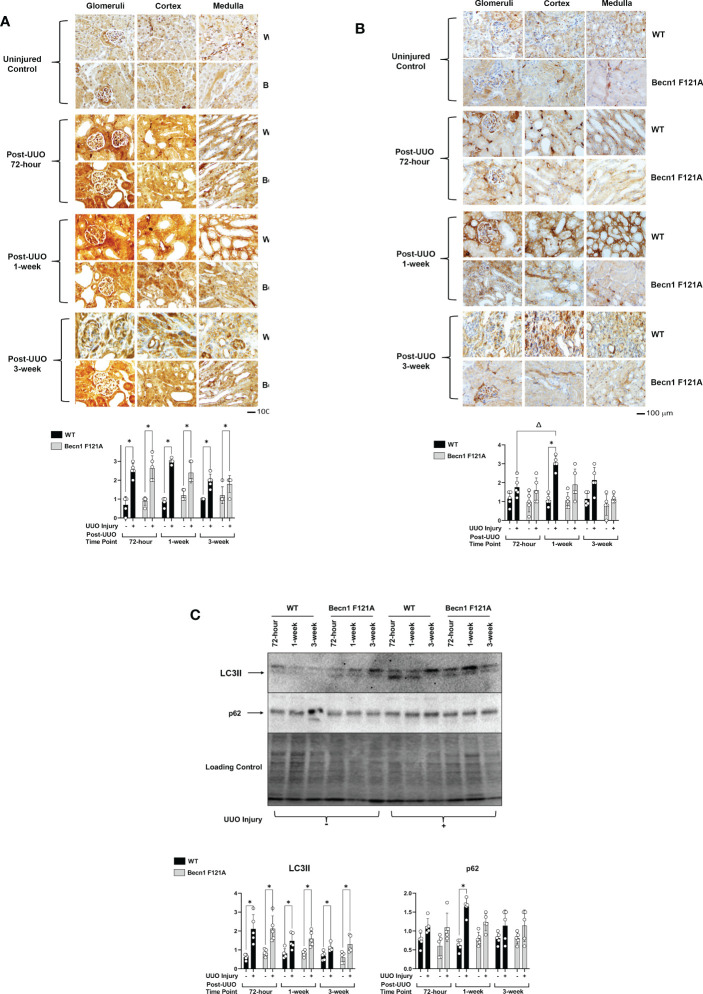
Autophagy flux in kidney during UUO and the effect of forced expression of Beclin-1 F121A mutation Mice of wild type (WT) and *Becn1* ^F121A/F121A^ strain (Becn1 F121A) were subjected to UUO injury as described, and kidneys were harvested at 72-hour, 1- and 3-weeks post-injury. Formalin- fixed tissue samples were examined by IHC using antibodies against LC3B **(A)** and p62 **(B)**. Quantification of signal intensity was performed by the ImageJ software based on images captured from at least ten sections per group. Threshold quantification was normalized to the uninjured control kidney. **(C)**. Kidney tissues were harvested, and total tissue lysates were prepared. Levels of LC3II and p62 were analyzed by western blots. The level of total protein signals per lane was served as a loading control. Results were quantified by densitometry and expressed as fold changes relative to controls. All data were expressed as mean ± standard derivation (SD) and analyzed by ANOVA with Tukey’s multiple-comparison test to determine significant differences between groups. Significant differences are shown as * for injured vs. uninjured controls and *Δ* for between time points (n= 5, *p ≤* 0.05).

IHC signal intensities in all kidney tissue regions, including the three main functional regions of kidney – cortex, glomeruli, and medulla, were quantified by ImageJ software. Individual areas of these three regions were shown in the IHC images presented in this report. Following UUO in WT mice, a significant surge of LC3II in the cortex, glomeruli, and medulla at all time points was seen (**Figure 1A**), and these changes in LC3II were accompanied by a drastic accumulation of p62, which peaked 1-week post-injury (2X increase over control; **Figure 1B**).

Mice with transgenic expression of an active mutant form of Beclin-1*, Becn1* ^F121A/F121A^, have a GOF mutation in Beckin-1 resulting in an up-regulation of autophagy (31). The boosted autophagy activity in this mouse strain has been demonstrated in multiple models (32, 37). To determine the role of Beclin-1-dependent autophagy in renal fibrosis, *Becn1* ^F121A/F121A^ mice underwent UUO as well. Increased expression of Becn1 did not affect UUO-triggered increases in LC3 (**Figure 1A**) but significantly limited p62 accumulation (**Figure 1B**), suggesting a positive support of a physiological flow of autophagy flux.

Using an alternative approach, we examined the status of autophagy flux in the same set of heart tissues by western blot analysis. Compared to shams, the level of LC3II was higher in all kidney samples subjected to UUO, whereas p62 was accumulated in kidneys of WT but not *Becn1* ^F121A/F121A^ mice 1-week post UUO injury (**Figure 1C**).

### Activation of Beclin-1 attenuates renal inflammation in response to UUO

**Figure 2:**
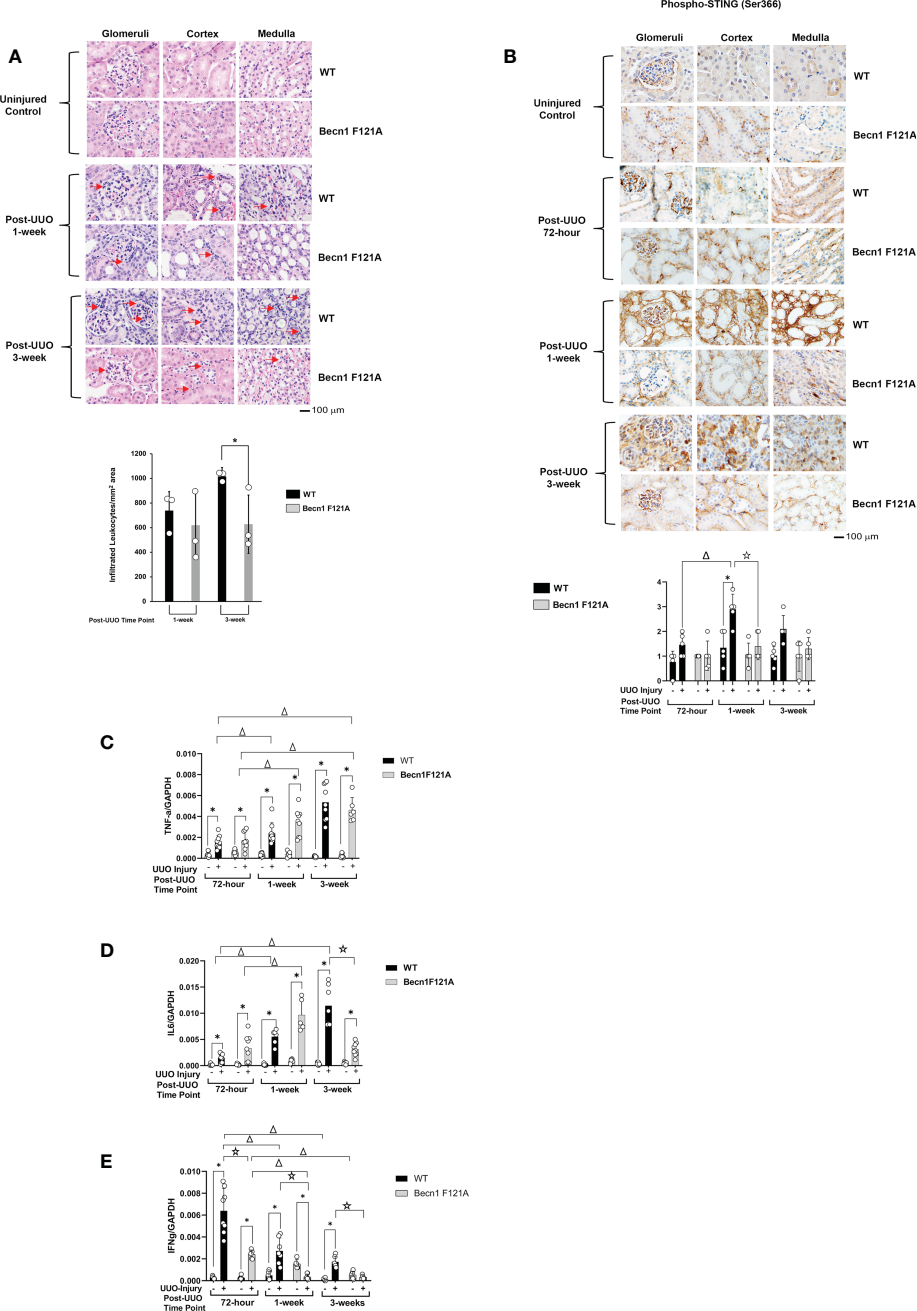
Forced expression of Beclin-1 F121A mutation limits renal inflammation during UUO Mice of wild type (WT) and *Becn1* ^F121A/F121A^ strain (Becn1 F121A) were subjected to UUO injury as described, and kidneys were harvested at 72-hour, 1- and 3-weeks post-injury. **(A)**. Kidney histology was evaluated by H&E staining using formalin-fixed tissue samples. Red arrows indicate accumulated infiltrated leukocytes. **(B)**. Tissue slides were examined by IHC using antibody against phosphorylated STING. In **(A, B)**, quantification of signal intensity was performed by the ImageJ software based on images captured from at least ten sections per group. Threshold quantification was normalized to the uninjured control [N=3 for **(A)** and 5 for **(B)**]. Expression levels of cytokines TNF-*α* **(C)**, IL6 **(D)**, and IFN*γ* **(E)** were detected from freshly frozen tissue by quantitative real-time PCR. Results were normalized using GAPDH as an internal control. Relative expression of target genes was calculated by the 2^-ΔΔCt^ method. Reactions were performed in triplicate. All data were expressed as mean ± SD and analyzed by ANOVA with Tukey’s multiple-comparison test to determine significant differences between groups. Significant differences are shown as * for injured vs. uninjured controls, *Δ* for between time points, and for WT vs. Becn1 F121A (*p ≤* 0.05).

Post-transplant inflammation secondary to rejection or infection has been shown to lead to tissue ischemia with the potential to result in concomitant fibrosis and scarring (38). To examine the effect of Beclin-1 expression on the injury-related inflammation, we compared H&E staining between WT and *Becn1* ^F121A/F121A^ mice following UUO. As shown in **Figure 2A**, in WT mice, accumulation of infiltrated leukocytes became evident in all kidney regions examined, including cortex, glomeruli, and medulla, starting at 1-week post UUO, and continued to be prominent 3-weeks post UUO. However, in *Becn1* ^F121A/F121A^ mice, leukocyte infiltration 1-week post UUO was decreased, especially

in cortex, when compared with the WT mice. Furthermore, 3-weeks post UUO, leukocyte infiltration continued to be decreased in *Becn1* ^F121A/F121A^ mice, indicating an attenuated inflammatory response.

We further examined whether Beclin-1 expression affected inflammatory factors that were previously identified to associate with renal failure and acute renal injury (AKI). Among a variety of inflammation networks, the cyclic GMP-AMP synthase (cGAS) stimulator of interferon genes (STING) signaling pathway was reported to be triggered by mitochondrial damage in AKI (39). In the UUO model, we evaluated the activation status of STING by assessing levels of STING phosphorylation at serine 366, a commonly known marker of STING activation (40). As summarized in **Figure 2B**, quantification of IHC intensity showed that UUO induced a 2-fold increase in the phosphorylation of STING in the renal cortex, glomeruli, and medulla in WT mice 1 week post injury and gradually declined 3 weeks following injury. However, in *Becn1* ^F121A/F121A^ mice, the level of phosphorylated STING responded little to UUO and remained lower than that in WT mice at all times examined.

Additionally, in the kidney tissues harvested post UUO, we examined the gene expression profiles of cytokines, TNF-*α*, IL6, and IFN*γ*, by quantitative real-time PCR analysis. We detected that UUO stimulated a progressive increase in TNF-*α* production in kidneys of both the WT and the *Becn1* ^F121A/F121A^ mice (**Figure 2C**). A similar trend was detected for the IL6 expression in WT mice but not *Becn1* ^F121A/F121A^ mice. In the latter case, the renal production of IL6 was initially increased but significantly declined 3 weeks post UUO; reduced to more than half amount of that in the WT mice (**Figure 2D**). Interestingly, the expression of IFN*γ* revealed a distinctly different pattern. In WT, the UUO injury stimulated a drastic elevation in IFN*γ* at the early phase, followed by a gradual decrease at later time points (**Figure 2E**). Though the expression of IFN*γ* followed the same pattern in *Becn1* ^F121A/F121A^ mice, its level was merely about 1/3 of that in WT at 72-hour post injury and decreased to the level of uninjured controls at 3-week post-injury. Taken all, our analysis of inflammatory factors suggests that promoting autophagy *via* Beclin-1 activation attenuated renal inflammation induced by UUO injury.

### Activation of Beclin-1 attenuates renal integrated stress response in response to UUO

**Figure 3:**
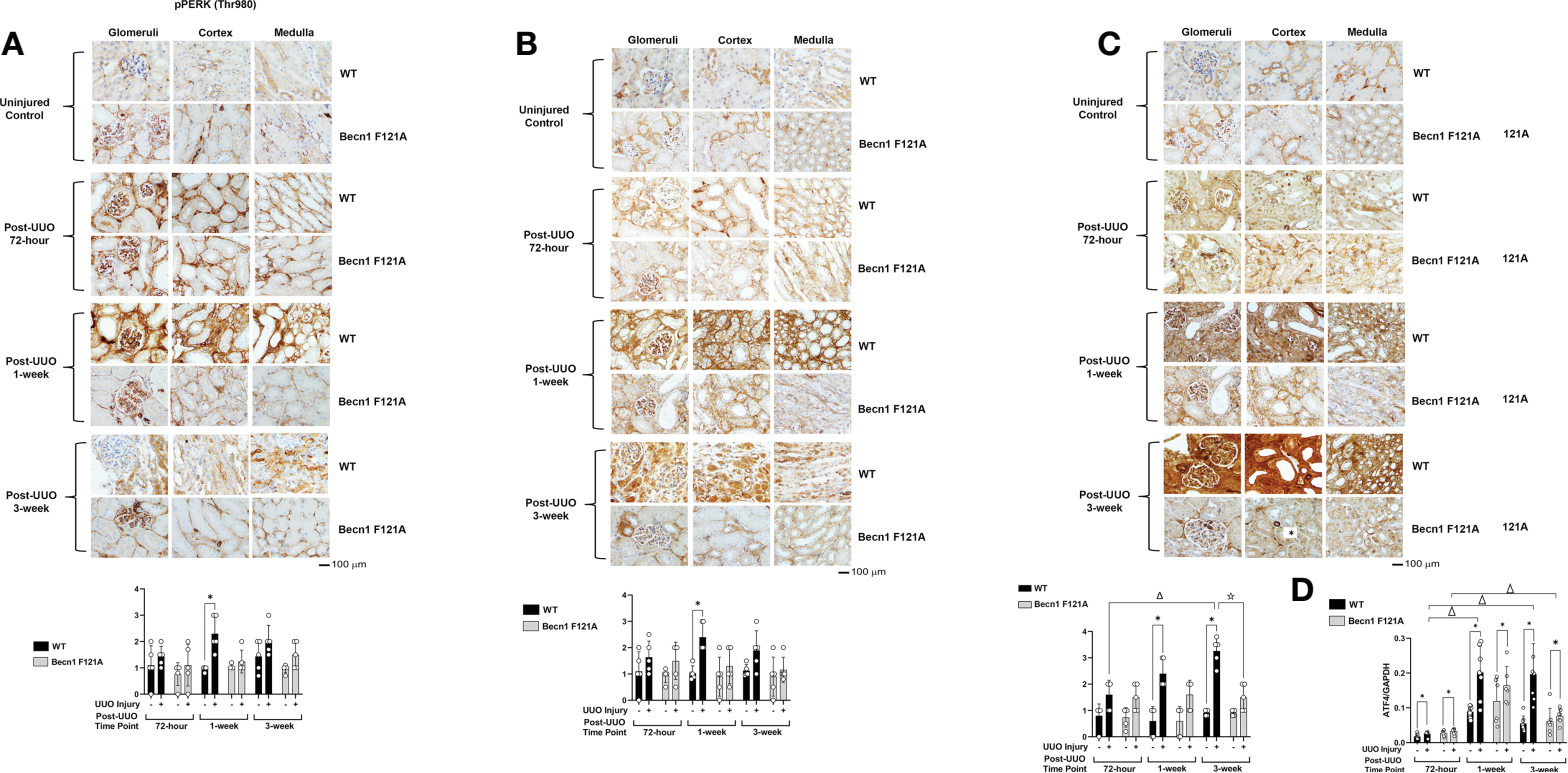
Forced expression of Beclin-1 F121A mutation attenuates renal ISR during UUO Mice of wild type (WT) and *Becn1* ^F121A/F121A^ strain (Becn1 F121A) were subjected to UUO injury as described, and kidneys were harvested at 72-hour, 1- and 3-weeks post-injury. Formalin-fixed tissue samples were examined by IHC using antibodies against phosphorylated PERK **(A)**, phosphorylated eIF2α **(B)**, and ATF4 **(C)**. Quantification of signal intensity was performed by the ImageJ software based on images captured from at least ten sections per group. Threshold quantification was normalized to the uninjured control (N=5). **(D)** ATF4 expression was detected from freshly frozen tissue by quantitative real-time PCR. Results were normalized using GAPDH as an internal control. Relative expression of target genes was calculated by the 2^-ΔΔCt^ method. Reactions were performed in triplicate. All data were expressed as mean ± SD and analyzed by ANOVA with Tukey’s multiple-comparison test to determine significant differences between groups. Significant differences are shown as * for injured vs. uninjured controls, *Δ* for between time points, and for WT vs. Becn1 F121A (*p ≤* 0.05).

Recent studies revealed that the integrated stress response (ISR) activated *via* the axis of protein kinase R-like endoplasmic reticulum (ER) resident kinase (PERK) and α-subunit of eukaryotic initiation factor 2 (eIF2α) signaling is an adaptive, protective mechanism in the heart underwent ischemia/reperfusion (I/R) injury (41). We went on to test the role of renal ISR under the condition of UUO injury. The activation status of ISR *via* PERK can be detected by the phosphorylation of PERK, phosphorylation of eIF2α, and the expression of activating transcription factor 4 (ATF4), a ISR effector protein (41). We compared these signals in the kidney tissues harvested at 72-hour, 1- and 3-week post UUO. By IHC staining with antibodies targeted specifically at the phosphorylation sites of PERK and eIF2α, we observed that UUO triggered a more than 1-fold increase in the phosphorylation of PERK and eIF2α in WT mice at 1-week post UUO, which was the highest among the three time points examined (**Figures 3A, B**). As hypothesized, the expression of ATF4 was gradually simulated by UUO; its protein level reached to an over 3-fold (**Figure 3C**) and mRNA an over 5-fold (**Figure 3D**) increases compared with uninjured controls respectively at 1-week and 3-week post injury. However, in *Becn1* ^F121A/F121A^ mice, no statistical differences between the UUO-injured and uninjured control were detected for the phosphorylation of either PERK or eIF2α (**Figures 3A, B**). Regarding ATF4 expression, though a notable increase in mRNA level was detected at 1-week post injury, Beclin-1 F121A mutation dramatically suppressed ATF4 mRNA expression at the time point of 3-week post UUO (**Figure 3D**). These data strongly suggest that, unlike the situation in the I/R heart, stimulation of renal ISR may lead to maladaptive signals during UUO injury, and enhancing autophagy possesses a potential to effectively alleviate this pathological response.

### Activation of Beclin-1 alleviates renal fibrosis in response to UUO

**Figure 4:**
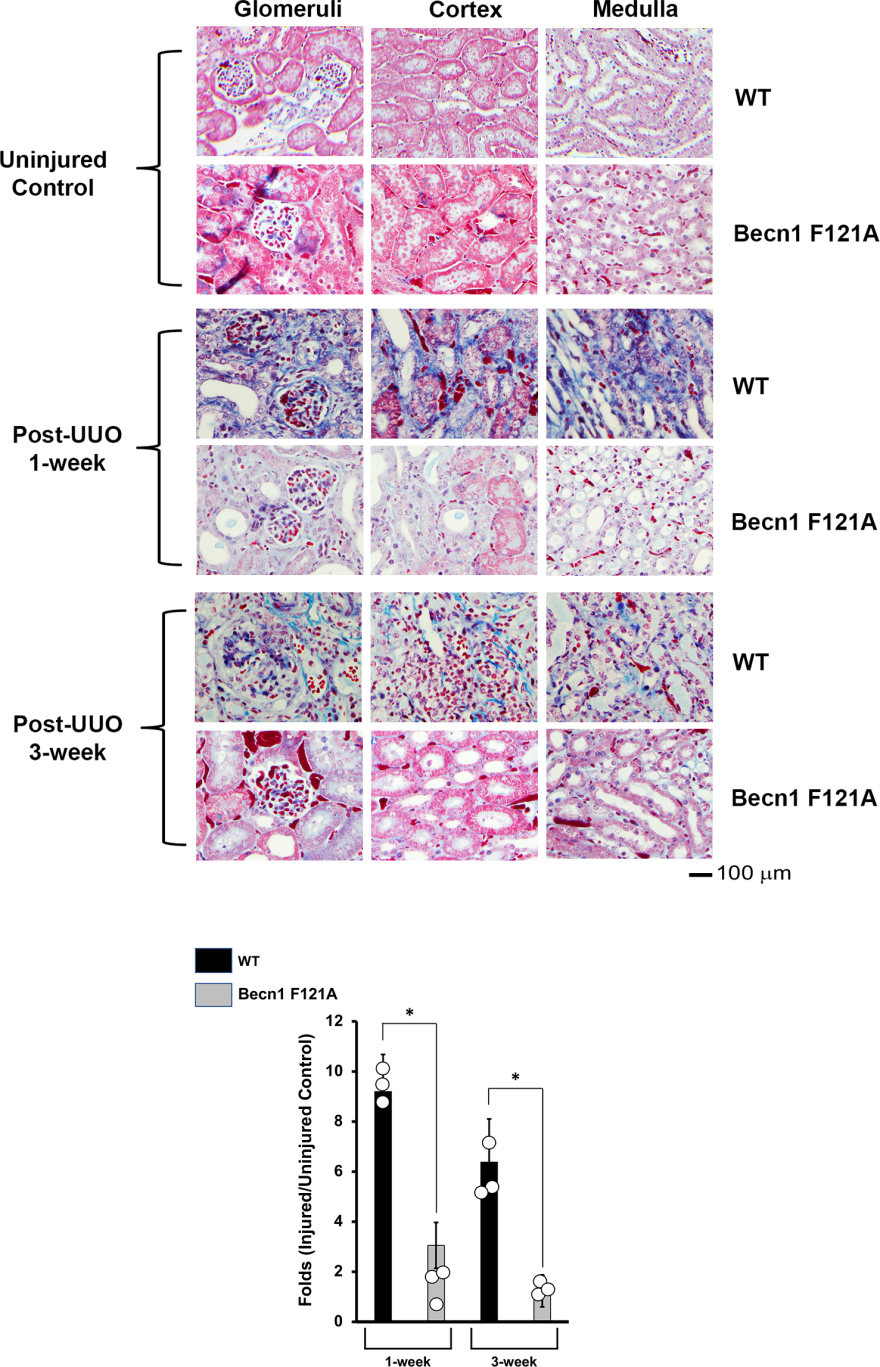
Forced expression of Beclin-1 F121A mutation alleviates renal fibrosis during UUO. Mice of wild type (WT) and *Becn1* ^F121A/F121A^ strain (Becn1 F121A) were subjected to UUO injury as described, and kidneys were harvested at 72-hour, 1- and 3-weeks post-injury. Formalin-fixed tissue samples were examined by trichrome stain. Quantification of signal intensity was performed by the ImageJ software based on images captured from at least ten sections per group. Threshold quantification was normalized to the uninjured control (N=3). All data were expressed as mean ± SD and analyzed by ANOVA with Tukey’s multiple-comparison test to determine significant differences between groups. Significant differences are shown as * for injured vs. uninjured controls, *Δ* for between time points, and for WT vs. Becn1 F121A (*p ≤* 0.05).

Ultimately, inflammation and ischemia/reperfusion injury lead to fibrosis, which is the main cause of renal graft loss following transplant. Our previous studies showed that, in mice subjected to UUO injury, a significant increase in interstitial collagen deposition developed 2 weeks post-UUO (29). By Masson’s Trichrome Staining, we compared levels of collagen disposition between WT and *Becn1* ^F121A/F121A^ mice following UUO (**Figure 4**). A staggering deposition of collagen fiber was detectable in all the examined renal regions of WT mice following UUO, including cortex, glomeruli, and medulla 1-week and 3-weeks post UUO. However, fibrosis development was much decreased in *Becn1* ^F121A/F121A^ mice following UUO, strongly suggesting that enhancing autophagy *via* Beclin-1 activation provides an effective control over renal fibrosis in response to injury.

## Discussion

Following renal transplant, Interstitial Fibrosis and Tubular Atrophy (IFTA) is recognized as a main mechanism for resulting in long-term graft failure (2). Therefore, it is critical to understand the molecular events that lead to IFTA and to identify novel therapeutic targets to improve long-term outcomes. Our previous investigations suggest that promoting autophagy *via* Beclin-1 factor is beneficial for improving organ function, mitigating inflammation, and alleviating fibrosis in models of sepsis-induced cardiomyopathy (21, 32, 42, 43). In this report we extrapolated previous data and applied it on a renal injury model. We examined whether Beclin-1 expression results in a similar protection in a mouse UUO model. UUO is a commonly used model for the studies of renal fibrosis. Even though the mechanism of injury is a result of increasing hydrostatic pressure, signaling cascades equal to those seen in I/R resulting the activation of pro- fibrotic pathways. Therefore, this model of injury can be used as a surrogate for renal I/R.

Our work showed that UUO caused a significant degree of blockage in renal autophagy flux. This was demonstrated by an increase in autophagosome formation, indicated by increases in LC3II, and an accompanied decrease in lysosomal degradation, shown by accumulated p62 (**Figure 1**). In addition, UUO stimulated renal inflammation, as demonstrated by leukocytic infiltration as well as activation of the STING inflammatory pathway, and with concomitant induction of cytokines such as TNFa, IL6 and IFNg (**Figure 2**). Interestingly, we detected a boosted activation of the ISR pathway in UUO-injured kidneys, given the strong evidence concluded from the activation of signaling factors PERK and eIF2α and the dramatically elevated expression of ISR effector ATF4 (**Figure 3**).

In comparison, a genetically modified mouse strain carrying a forced expression of active mutant Beclin-1, *Becn1* ^F121A/F121A^, resulted in alleviated obstruction of autophagy, reduced inflammation *via* a differential control over inflammatory factors such as STING, IL6 and IFNg, and effectively suppressed ISR in kidneys subjected to UUO (**Figures 1**–**3**). Importantly, we also observed that the development of fibrosis following UUO was largely attenuated by introducing the expression of active mutant Beclin-1 (**Figure 4**). Based on these new findings, we propose that promoting Beclin-1-dependent autophagy attenuates the inflammatory cascade associated with renal injury. Our results also provide evidence that ISR is also significantly attenuated following UUO in the setting of Beclin-1 expression.

Previous studies have demonstrated that autophagy is involved in the pathological conditions of both acute and chronic renal failure (44). Like in other organs such as the heart, basal autophagy is essential to the maintenance of metabolic homeostasis, cellular and tissue structure, and physiological function, whereas excessive or insufficient autophagy induction can lead to worsening disease progression or an inability to protect against progression of injury (21, 43, 45). In acute kidney injury, autophagy is initially induced as a protective mechanism, however persistent activation of autophagy becomes pathological, eventually contributing to the stimulation or under-protection of pro-fibrotic mediators (46). Our studies showed that autophagy provides protection from the development of fibrosis, consistent with previously published results (47). Published data also showed that autophagy was induced in the obstructed kidney in a time-dependent manner, increased initially and declining at later (48). Our data did not reveal signs of autophagy at all time points examined (**Figure 1**), however, we cannot exclude the possibility that autophagy was induced at an earlier time point.

We also want to point out that the role of autophagy in the development of renal fibrosis may hinge upon the response of various cell types to ischemic injury (49). Data on the role of individual cell types has shown that podocytes, renal tubular epithelial cells, as well as endothelial cells all undergo autophagy which can be ultimately associated with fibrosis. Though there is an immune response secondary to ischemic injury, most of the autophagic pathways are found localized to parenchymal cells. Podocytes localized to glomeruli have been shown to undergo autophagy secondary to renal injury (50). Endothelial cells in the renal parenchyma have also been shown to undergo autophagy leading to filtration loss and fibrosis (51). Finally, renal tubular epithelial cells, the most common cell type in kidneys, have been shown in multiple studies to undergo autophagy secondary to ischemic injury leading to the initiation of repair mechanisms (52, 53). However, accumulative evidence supports the notion that, maladaptive autophagy, either insufficient or excessive, as well as continuous bouts of injury facilitate the development of fibrosis. Our results suggest that, while UUO injury obstructs a physiological autophagic flux essential for renal physiology, promoting autophagy *via* autophagy initiation factor Beclin-1 will alleviate this pathological process. Additionally, we are aware that published evidence showed that uncontrolled elevation of autophagy induces autophagy-dependent cell death, autosis (54), which might be a potential factor that promotes fibrosis. However, in our experimental setting, signs of cell death were not detectable in the kidney tissue of *Becn1* ^F121A/F121A^ mice with or without UUO injury. At the current stage, it remains unclear whether this form of cell death happens in a cell- or tissue-specific fashion in kidney tissue. Most likely, if occurred, an autophagy level may need to meet certain threshold to trigger the response, which could be above and beyond the autophagy level offered by Beclin-1 mutation F121A. Our future studies will attempt to address the specific nature of cell-type dependent Beclin-1 expression and function, especially in renal parenchymal cells, in response to ischemic injury.

Our study further provided evidence elucidating that Beclin-1 expression alleviates renal fibrosis following UUO. The mechanism by which Beclin-1 ameliorates fibrosis involves the regulation of downstream signals of inflammation and ISR. Inflammatory cGAS- STING pathway triggered by mitochondrial damage and mitochondrial metabolic deficiencies was previously demonstrated as a cause for stimulating renal inflammation and fibrosis, as mtDNA released from damaged mitochondria served as ligands stimulating cGAS-STING cascade in tubular cells (39, 55). The data presented in this report showed Beclin-1 dependent control of STING activation in UUO-injured kidney (**Figure 2B**), presumably a result from autophagy-induced improvement in mitochondrial quality control. Our previous investigation in a mouse model of endotoxemia demonstrated that Beclin-1 possesses the capability to prevent mitochondrial structural damage, trigger adaptive mitophagy, and thus reduce the amount of free mtDNA in myocardium (21). This anti-inflammatory pathway may be at play in protecting injured kidneys from ultimately developing fibrosis. We also examined production of cytokines in our study and found that activation of Becin-1 provided a significant reduction on levels of IFN*γ* and IL-6 at late time point (3-weeks) following UUO but had no effect on TNF-*α* (**Figures 2C, D, E**). The control of cytokine production *via* Beclin-1 is likely due to autophagy-mediated improvement in cellular physiology and survival, which minimizes the generation of danger-associated molecular patterns (DAMPs) caused by the injury. However, this Beclin-1 dependent effect on cytokines does not seem universal, which may be due to the fact that cytokines are generated from different cell types with different time dynamics. Therefore, a further detailed, cell type-dependent analysis in UUO-injured kidney is warranted to understand the role of Beclin-1 in control of inflammation. Additionally, the types, ratios, and activation state of infiltrated immune cells during the injury also awaits to be characterized.

In this study, we found that UUO stimulated a dramatic elevation in renal ISR (**Figure 3**). Like autophagy, ISR is a pro-survival mechanism that is stimulated to retaliate challenges from severe stress. However, responses under or over properly needed levels of ISR are maladaptive and can lead toward cell death (30, 56, 57). The central point of ISR is the phosphorylation of eIF2α by members of eIF2α kinase family such as PERK, followed by a diminished global protein synthesis but a stimulated expression of certain selected genes such as ATF4, an effector of ISR. PERK (also named EIF2AK3), a single-pass transmembrane protein located in the ER membrane, functions as an eIF2α kinase when encountered ER stress (58). In our experiment setting, significant increases in phosphorylation of PERK and eIF2α were detected in kidneys at 1-week post UUO (**Figures 3A, B**), followed by a surge in ATF4 expression at 3-week post the injury (**Figures 3C, D**). The response of renal ISR following UUO is likely a result of ER stress. ER stress has been previously identified to be associated with renal fibrosis in UUO and other models of kidney disease (59, 60). Nonetheless, in mice carrying active mutant Beclin-1, these ISR mediators were diminished to physiological levels following UUO. It has been previously hypothesized that the low induction of ER stress is used as an adaptive mechanism known as preconditioning, whereas uncontrolled ER stress delivers the opposite effect. Our data suggest that Beclin-1-dependent autophagy limits the activation of overwhelming, maladaptive ISR in the kidney, showing a protective effect. Our recent published study revealed that Beclin-1 supports the function and quantity of mitochondria-associated membranes (MAMs), a subcellular domain bridging mitochondria and ER, in the heart under the pathological condition of endotoxemia (42). Therefore, Beclin-1 may function as a pivotal regulator for controlling the crosstalk between mitochondria and ER and therefore toning responses of inflammation.

Afterall, our data showed that Beclin-1 activation reduced renal fibrosis in UUO (**Figure 4**), which is in concurrence of the recent publication that evaluated the same mouse strain (24). Our study further argues Beclin-1 for a global control of renal response to inflammatory injury which if increased may protect against downstream mediators ultimately leading to renal fibrosis. Furthermore, our presentation of Beclin-1 to control renal fibrosis *via* distinctively mitigating inflammatory responses and effectively extenuating ISR which is the first report of this observation in literature to our knowledge. This study also suggests that, in addition to Beclin-1, signaling molecules in pathways of ISR and cGAS-STING may become promising therapeutic targets for renal fibrosis. Future studies will aim to decipher the signaling cascade led by Beclin-1 in the process of fibrosis and to identify potential therapies for clinical usage.

## Data availability statement

The raw data supporting the conclusions of this article will be made available by the authors, without undue reservation.

## Ethics statement

The animal study described in this study was reviewed and conducted under the oversight of Loyola University Chicago Institutional Animal Care and Use Committee and conformed to the “Guide for the Care and Use of Laboratory Animals” by National Research Council (US) when establishing animal research standards.

## Author contributions

RL-S and QSZ conceived the project, designed the study, and wrote the manuscript. AN, MK, SK, LS, and XD conducted the experiments and contributed to the data analysis. All authors contributed to the article and approved the submitted version.
